# On usage of artificial intelligence for predicting mortality during and post-pregnancy: a systematic review of literature

**DOI:** 10.1186/s12911-022-02082-3

**Published:** 2022-12-19

**Authors:** Elisson da Silva Rocha, Flavio Leandro de Morais Melo, Maria Eduarda Ferro de Mello, Barbara Figueiroa, Vanderson Sampaio, Patricia Takako Endo

**Affiliations:** 1 Programa de Pós-Graduação em Engenharia da Computação, Universidade de Pernambuco, Recife, Brazil; 2 Universidade Federal de Pernambuco, Recife, Brazil; 3 Programa Mãe Coruja Pernambucana, Secretaria de Saúde do Estado de Pernambuco, Recife, Brazil; 4 Instituto Todos pela Saúde, São Paulo, Brazil

**Keywords:** Machine learning, Deep learning, Stillbirth, Neonatal mortality, Infant mortality

## Abstract

**Background:**

Care during pregnancy, childbirth and puerperium are fundamental to avoid pathologies for the mother and her baby. However, health issues can occur during this period, causing misfortunes, such as the death of the fetus or neonate. Predictive models of fetal and infant deaths are important technological tools that can help to reduce mortality indexes. The main goal of this work is to present a systematic review of literature focused on computational models to predict mortality, covering stillbirth, perinatal, neonatal, and infant deaths, highlighting their methodology and the description of the proposed computational models.

**Methods:**

We conducted a systematic review of literature, limiting the search to the last 10 years of publications considering the five main scientific databases as source.

**Results:**

From 671 works, 18 of them were selected as primary studies for further analysis. We found that most of works are focused on prediction of neonatal deaths, using machine learning models (more specifically Random Forest). The top five most common features used to train models are birth weight, gestational age, sex of the child, Apgar score and mother’s age. Having predictive models for preventing mortality during and post-pregnancy not only improve the mother’s quality of life, as well as it can be a powerful and low-cost tool to decrease mortality ratios.

**Conclusion:**

Based on the results of this SRL, we can state that scientific efforts have been done in this area, but there are many open research opportunities to be developed by the community.

## Introduction

Pregnancy has its natural physiological path for a healthy baby to have its life started. However, when this path is discontinued due to a stillbirth, it may impact negatively on the quality of life of all individuals related to the misfortune, involving physical, psychological, economic and/or social aspects. Furthermore, the stillbirth rate is also a sensitive indicator that reflects on socioeconomic conditions and is related to the quality of prenatal care and care during pregnancy [1, 2].

In 2020, an estimated two million pregnancies were not completed due to stillbirths. Among these, more than 40% of deaths occurred during the labor [1]. In the same year, 2.4 million children died in the first 28 days (neonatal mortality), representing 47% of all deaths of children under 5 years old. In 2019, about 1 million newborns died in the first 24 h (early neonatal mortality) [3], and approximately 1.6 million babies aged between 28 and 365 days died (infant mortality) [4]. Causes of death in the first weeks include low birth weight, infections, neonatal asphyxia and complications of preterm birth. The lack or poor quality of maternal health care services during childbirth contributes to causes of death. Furthermore, the absence of prenatal care interventions and prevention of maternal complications before delivery corroborate these data [2, 5].

Preterm birth and neonatal death are inversely connected. The lower the gestational age of the newborn, higher the risk of death leading to greater attention to preterm births [2]. Some risk factors related to the mother such as age, smoking, diabetes, hypertension, fetal anomaly and miscarriages increase the chances of premature birth [2, 6].

The 2030 Agenda [7] proposed by the Organization of the United Nations (UN) predicts the reduction of neonatal mortality and mortality of children under 5 years of age in its Sustainable Development Goals (SDGs). However, specific targets aimed at reducing of fetal mortality were absent from the Millennium Development Goals (MDGs) [8] and were not covered by the Agenda 2030. Unfortunately, this public health issue has been overlooked, and stillbirths have been largely absent from tracking health data around the world, hiding the true extent of this problem.

Given this context, public policies for maternal and child health are essential to prevent these deaths. It is possible to improve the quality of services provided in order to end preventable stillbirths and achieve good quality of health in newborns, with good antenatal care, specialized care in childbirth, postpartum care and especially, care for small and sick newborns [1, 3].

Recent studies demonstrate that Artificial Intelligence (AI), particularly through machine learning and deep learning models, offers considerable potential to predict prematurity, birth weight, mortality, hypertensive disorders, postpartum depression, among others [9]. Machine learning is also being used to identify risks of perinatal mortality

[10–13] and fetal death [14, 15]. As they have feasible operational costs, which makes it easier to be implemented, these computational tools can also be a valuable ally, especially for nations with limited resources.

We found only one systematic literature review (SLR) that addressed stillbirths, perinatal mortality, neonatal mortality, and infant mortality using these AI techniques, published in 2021 by Mangold et al. [16]. They focused on works for predicting neonatal mortality. In contrast, in this SLR, our interest is to evaluate in the state-of-the-art on works that proposed machine learning and deep learning models to classify stillbirth, perinatal, neonatal and infant deaths. Hereafter, whenever we mention mortality, please consider stillbirth, perinatal mortality, neonatal mortality and infant mortality.

## Material and methods

As discussed earlier, stillbirth is a real public health concern around the world, and the development of AI based solutions is becoming an open field for research with many challenges. This SLR becomes necessary to understand at what point AI has contributed to detect risk and undesirable outcomes for pregnancy, and also to understand how the progress is in aspects related to stillbirths, such as neonatal, perinatal and infant mortality. The main goal of this work is to answer the following research questions (RQ):

- What types of mortality are the focus of researches that used machine learning and deep learning?
- What data is being used in researches on classification of mortality?
- What machine learning and deep learning techniques are being used in researches related to the classification of mortality?
- How is the performance of machine learning and deep learning models evaluated in the classification of mortality?

The methodology used to guide this SLR is based on the PRISMA statement, conformed to its checklist available at https://prisma-statement.org/. We used this methodology to find works that addressed the use of machine learning and/or deep learning in the context of mortality.

### Data sources and searches

We considered the following databases as the main sources for our research: IEEE Xplore^1^, PubMed^2^, ACM Digital Library^3^, Springer^4^ and Scopus^5^.

The collection of primary studies was done through searches in the databases, using the following search string: ((“deep learning” OR “machine learning”) AND (“stillbirth” OR “fetal death” OR “infant death” OR “neonatal mortality” OR ”neonatal death” OR “perinatal”) AND (“prediction” OR “classification”)) IN (Metadata) OR (Title) OR (Abstract).

### Eligibility criteria

As we can find many papers that are not strictly related to our RQ (or can not answer our research questions), we defined some inclusion and exclusion criteria.

The works must explicitly present abstract computational models to classify or classify mortality risks, use at least one real database and be from the last 10 years (between 2012 and 2021). We remove works that are duplicates, unavailable or not in English, poster, tutorial or editorial works, and secondary or tertiary works.

### Studies selection

Three reviewers (ESR, FLMM and PTE) were responsible for identifying eligible works independently. When any disagreement came up, a fourth reviewer (VS) was consulted to reduce the risk of bias. At first, the title and abstract were screened and, after that, works retained went to a full-text reading. Lastly, works that passed the inclusion and exclusion criteria were selected for data extraction.

### Data extraction

Works were evaluated considering their quality, considering these seven quality questions were defined:

- Does the study make clear what its objectives are?
- Does the study describe the entire methodology used?
- Does the study describe the database used and the pre-processing performed (when necessary)?
- Does the study describe the configurations of the proposed models?
- Does the study describe how it arrived at the proposed models?
- Does the study clearly describe the results?
- Does the study make a good discussion based on the results?

For each quality question, the possibles answers and scores were: Yes (1 point), Partially (0.5 point), and No (0 point). Therefore, each study was graded with a score based on answers of each question. Studies that presented at least half (3.5) of the maximum score (7.0) were accepted for reading and further analysis.

After reading the 18 primary studies, we extracted information from each work, based on general characteristics of the study, methodology, dataset, models, models’ performance, challenges and limitations in order to answer the research questions previously established.

**Fig. 1:**
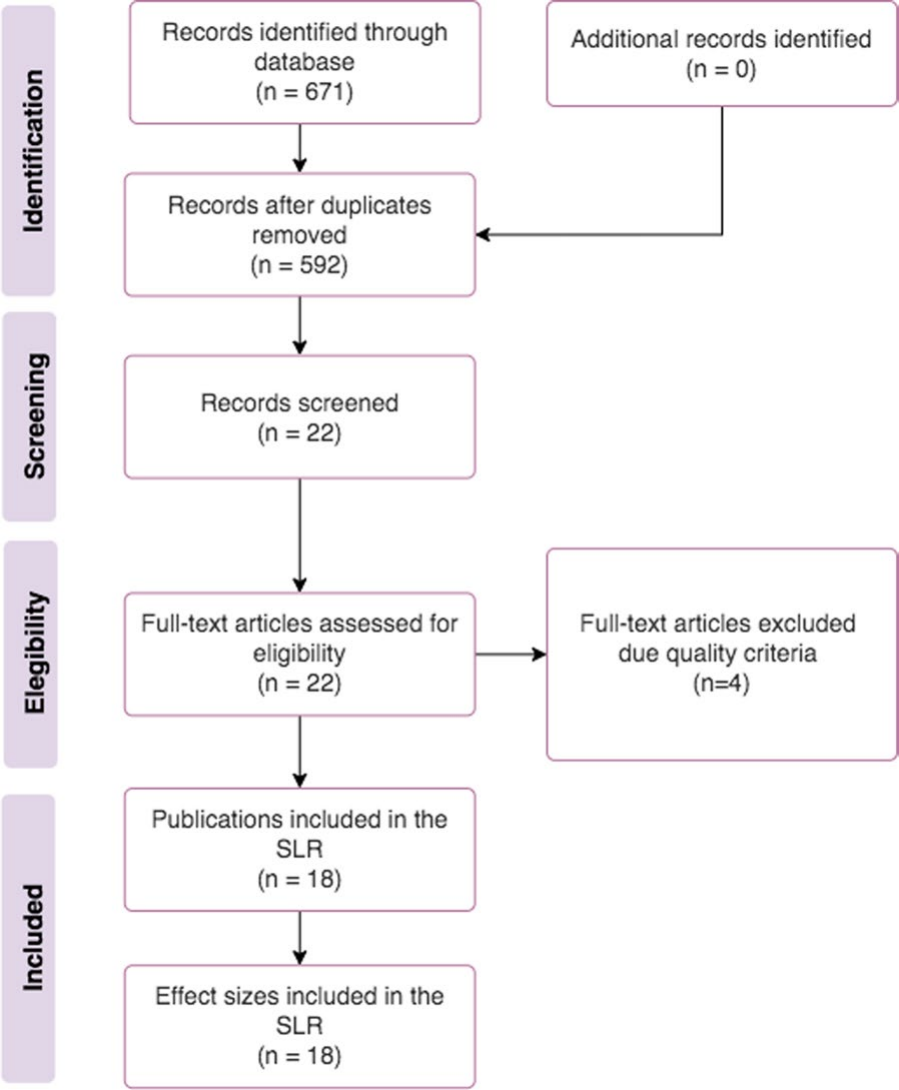
PRISMA flowchart of the review process

Figure 1 presents the PRISMA flow diagram used to summarize the works identified and those excluded due duplication or quality criteria.

## Results

### Descriptive analysis

In November 2021, the search returned 29, 80, 54, 104, and 404 works from IEEE Xplore, PubMed, ACM Digital Library, Scopus, and Springer, respectively, totaling 671 works. After removing duplicates, we read all abstracts applying the inclusion and exclusion criteria and then 22 works were selected. After the quality assessment, we finally obtained the 18 primary works for reading and extraction of information.

Even with the large number of works found in Springer and Scopus, only 1 and 2 of them were selected from these sources, respectively. PubMed was the source with the most primary works, 13 out of 18. The other 2 works were from IEEE, while ACM had no works selected.

The search for this SLR was restrict between the years 2012 to 2021, but the first work appeared in 2014. Of the primary works, 84% were published in the last three years: three in 2019, six in 2020 and six in 2021. This is a clear indication that AI still has a long way to go and good opportunities to develop scientific solutions for mortality prediction.

### What types of mortality are the focus of researches that used machine learning and deep learning?

**Fig. 2:**
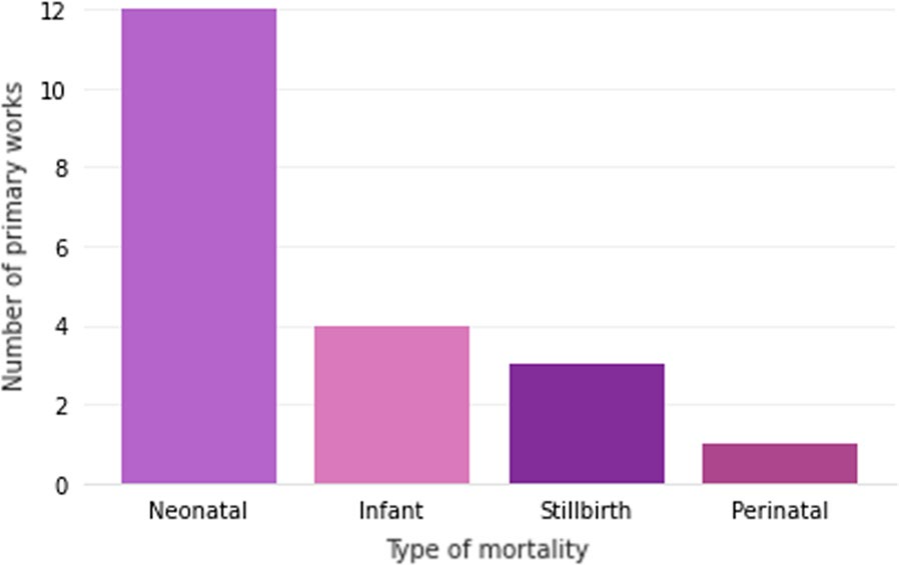
Number of selected works by type of mortality

In this SLR, we are focused on studies that used machine learning and deep learning models to classify some types of mortality, such as stillbirth, perinatal, neonatal and infant. Figure 2 shows the amount of work by type of mortality.

**Fig. 3:**
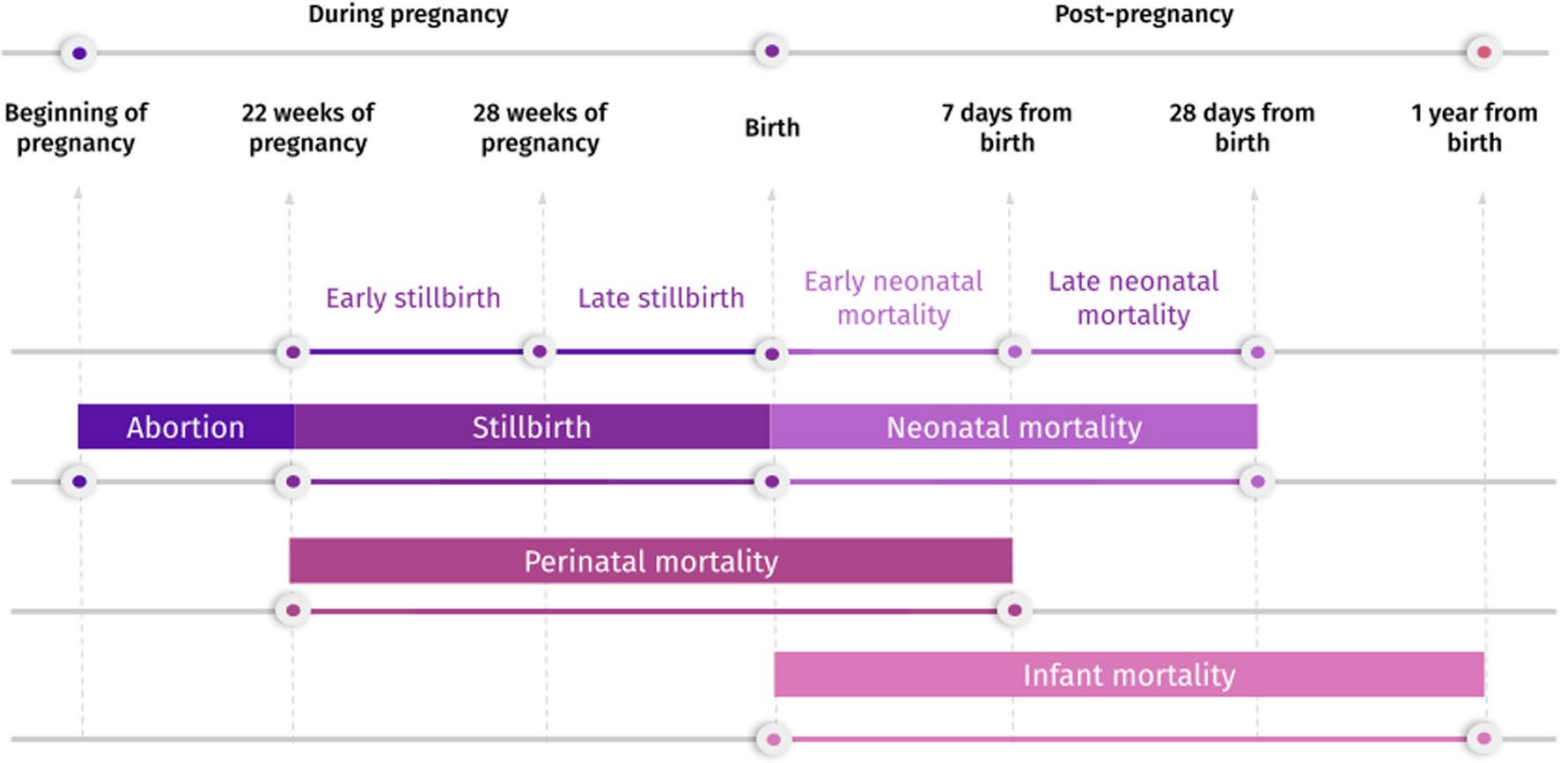
Definition of deaths that occur during or post-pregnancy up to 1 year from birth

The definition of stillbirth is not well established globally. The World Health Organization (WHO) recommendation defines fetal death as all deaths that occur after the 28th week of gestation or with a weight above 1000g; while intrauterine deaths occur during labor [17]. However, many countries use the definition of fetal death based on the 10th revision of the International Classification of Diseases (ICD-10), which considers deaths that occur with a gestational age greater than 22 weeks, or with a weight greater than 500g, or height greater than 25 cm, including deaths during labor [18, 19]. This lack of a universal definition implies inaccurate comparisons when there is a need to use national and international reporting data together. The works that used stillbirth classification used the ICD-10 rules. Figure 3 shows the definitions used in this SLR for deaths that occurred during pregnancy or up to 1 year after birth.

Most of the works are focused on neonatal mortality, 66% of the chosen works. Neonatal mortality is categorized when the neonate dies between his/her birth (when vital signs are detected after delivery) and the twenty-eighth day of his/her life. Many works focus on this stage because they can detect mortality based on comorbidity generated during the pregnancy or postpartum. Baker et al. [20], Cerqueira et al. [21], Sheikhtaheri et al. [22], Sun et al. [23] and Hsu et al. [24] classify the mortality of babies that after their birth were referred to the Neonatal Intensive Care Unit (NICU); and Podda et al. [25], Jaskari et al. [26] and Lee et al. [27] rank the probability of death in premature babies; and Cooper et al. [28] classifies post-operative newborn mortality.

There are also works that are related to infant mortality (4 works), stillbirth (3 works), and perinatal mortality (only 1 work). Infant mortality refers to deaths occurring between 29 days of life and 365 days (1 year from birth); and perinatal is the period from stillbirth to early neonatal, until the 6th day of life, as shown in Fig. 3.

Four primary studies carried out research that focused on more than one type of mortality: Valter et al. [29], Saravanou et al. [30] and Batista et al. [31] who worked with neonatal and infant deaths; and Shukla et al. [10] who studied the spheres of stillbirths and neonates.

### What data is being used in researches on classification of mortality?

A summary of data sets found in the studies of this SLR is available in the Table 1, describing the location where the data were collected, number of records, total attributes of the original data set, number of attributes used for the model training, attribute selection technique, data balance and problems with missing data.

**Table 1:** Summary of the data sets

Work	Location	Number of records	Number of attributes	Attributes used for training	Attribute selection	**Balancing**	**Missing data**
Valter et al. [29]	Brazil	6241	27	26	Yes	The data set is balanced	Yes
Hajipour et al. [32]	Iran	2386	Not described	16	Not specified	The data set is balanced	No
Saravanou et al. [30]	USA	12,000,000	128	128; 2; 1; 1	No	The original data set is imbalanced, but it was balanced	No
Baker et al. [20]	Israel	2751	Not described	17	No	The original data set is imbalanced, but it was balanced	No
Cerqueira et al. [21]	Brazil	293	114	4	Yes	The data set is imbalanced	No
Shukla et al. [10]	India; Pakistan; Congo; Zambia; Kenya; Guatemala	588,272	Not described	31	Yes	The data set is imbalanced	Yes
Malacova et al. [14]	Australia	960,745	Not described	Not described	Not specified	The data set is imbalanced	No
Sheikhtaheri et al. [22]	Iran	1762	Not described	17	Yes	The original data set is imbalanced, but it was balanced	Yes
Podda, et al. [25]	Italy	29,557	Not described	13	Yes	The data set is imbalanced	Yes
Batista et al. [31]	Brazil	1,135,444	23	23	No	The data set is imbalanced	No
Jaskari et al. [26]	Finland	977	Not described	Not described	Yes	The original data set is imbalanced, but it was balanced	Yes
Mboya et al. [12]	Tanzania	42,319	32	20	Yes	The original data set is imbalanced, but it was balanced	Yes
AlShwaish et al. [33]	USA	172,278	Not described	Not described	Not specified	The original data set is imbalanced, but it was balanced	Yes
Sun et al. [23]	India	757	Not described	49	No	The original data set is imbalanced, but it was balanced	Yes
Koivu et al. [15]	USA	12,867,146	26	17; 14; 25	Yes	The data set is imbalanced	No
Lee et al. [27]	USA	31,287,801	Not described	34	No	The original data set is imbalanced, but it was balanced	Yes
Cooper et al. [28]	USA; Canada	6499	Not described	68	Yes	The data set is imbalanced	Yes
Hsu et al. [24]	Taiwan	1734	Not described	41	Not specified	The data set is imbalanced	Yes

*Approximate value

**When the work used a number of different attributes

#### Data set size and balancing

The largest data set was used by Lee et al. [27] with over 31 million records, followed by Koivu et al. [15] and Saravanou et al. [30] with approximately 12 million each. These three largest data sets collected data from the United States of America (USA). The smallest data sets were used by Cerqueira et al. [21] from Brazil, Sun et al. [23] from India and Jaskari et al. [26] from Finland with 293, 757 and 977 records, respectively.

Although Lee et al. [27], Koivu et al. [15] and Saravanou et al. [30] used the largest data sets, the majority class represented more than 99% of the data, according to Table 2. Another thirteen works also suffer from imbalanced data set problems.

**Table 2:** Distribution of samples per classes

Work	Mortality	Classes	Samples	Proportion*
Valter et al. [29]	Neonatal	Neonatal mort.	657	0.491
		Non-neonatal mort.	682	0.509
	Infant	Infant mort.	911	0.489
		Non-infant mort.	952	0.511
Hajipour et al. [32]	Infant	Infant mort.	1076	0.451
		Non-infant mort.	1310	0.549
Saravanou et al. [30]**	Infant	Infant mort.	83,000	0.007
		Non-infant mort.	12,000,000	0.993
	Neonatal/Infant	Died < 1 h	–	–
		Died 1–23 h	–	–
		Died 1–6 days	–	–
		Died 7–27 days	–	–
		Died 28–365 days	–	–
		Non-infant mort.	–	–
Baker et al. [20]	Neonatal	Neonatal mort.	28	0.010
		Non-neonatal mort.	2723	0.990
Cerqueira et al. [21]	Neonatal	Neonatal mort.	39	0.133
		Non-neonatal mort.	254	0.867
Shukla et al. [10]	Stillbirth	Stillbirth	15,322	0.030
		Non-stillbirth	487,326	0.987
	Neonatal	Neonatal mort.	6268	0.013
		Non-neonatal mort.	481,058	0.987
Malacova et al. [14]	Stillbirth	Stillbirth	6836	0.007
		Non-stillbirth	953,909	0.993
Sheikhtaheri et al. [22]	Neonatal	Neonatal mort.	138	0.078
		Non-neonatal mort.	1624	0.922
Podda et al. [25]	Neonatal	Neonatal mort.	3570	0.121
		Non-neonatal mort.	25,987	0.879
Batista et al. [31]	Neonatal	Neonatal mort.	7282	0.006
		Non-neonatal mort.	1,128,162	0.994
	Infant	Infant mort.	10,902	0.010
		Non-infant mort.	1,124,542	0.990
Jaskari et al. [26]	Neonatal	Neonatal mort.	63	0.064
		Non-neonatal mort.	914	0.936
Mboya et al. [12]	Perinatal	Perinatal mort.	1561	0.037
		Non-perinatal mort	40,758	0.963
AlShwaish et al. [33]	Infant	Minor	167,026	0.970
		Moderate	2988	0.017
		Major	1529	0.009
		Extreme	735	0.004
Sun et al. [23]	Neonatal	Neonatal mort.	15	0.020
		Non-neonatal mort.	742	0.980
Koivu et al. [15]	Stillbirth	Early stillbirth	7924	0.001
		Late stillbirth	8310	0.001
		Non-stillbirth	11,907,611	0.999
Lee et al. [27]	Neonatal	Neonatal mort.	97,200	0.003
		Non-neonatal mort.	31,190,601	0.997
Cooper et al. [28]	Neonatal	Neonatal mort.	232	0.036
		Non-neonatal mort.	6267	0.964
Hsu et al. [24]	Neonatal	Neonatal mort.	278	0.160
		Non-neonatal mort.	1456	0.840

*Numbers were rounded

**Approximate value

According to Ramyachitra et al. [34], “*a two-class data set is implicit to be imbalanced when one of the classes in the minority one is heavily under-represented in contrast to the other class in the majority one*”. The imbalanced data set is a crucial challenge because the absent of solving this issue can lead classifiers to be biased towards the majority class.

From works with imbalanced data set, eight of them kept the data set as it is, while eight performed some balancing technique in order to cover this problem [12, 20, 22, 23, 26, 27, 30, 33]. The most common approaches used to balance a data set were: random oversampling (ROS) and random undersampling (RUS) [35].

Saravanou et al. [30], Baker et al. [20], Jaskari et al. [26], Alshwaish et al. [33], Sun et al. [23] and Lee et al. [27] applied the RUS technique, in which they re-sampled the data set based on the minority class; to do this, the majority class is cut randomly until it gets the same size of the minority class [36].

Sheikhtaheri et al. [22] and Mboya et al. [12] used a classic ROS technique, named Synthetic Minority Oversampling Technique (SMOTE), in which the majority class is kept as original and the minority class is randomly increased with synthetic data.

Sheikhtaheri et al. [22] created four different data sets using the SMOTE technique, varying the ratio of classes; and they also created a data set using the ADASYN technique, in which a weighted distribution of the minority class is used and samples that are harder to learn are prioritized.

Of the three largest data sets mentioned above, only Koivu et al. [15] performed training with an imbalanced data set, making it the largest data set used for model training, followed by Batista et al. [31] and Malacova et al. [14] with approximately 1 million records. However, these three works did not use balanced data, which can lead to problems in training the models and, therefore, it is important to analyze the evaluation metrics used by authors in order to present a fair comparison (see Section for details about metrics).

Regarding the smallest data sets, only Cerqueira et al. [21] did not perform the balancing and used all 293 data for training and testing, while Sun et al. [23] and Jaskari et al. [26] even with small databases, performed data balancing for training. On the other hand, the proportion of the majority class of the two works that performed the balancing was 0.98 and 0.936, respectively, and Cerqueira et al. [21] was 0.867.

#### Missing data

According to Phung et al. [37], “*missing data is a frequent occurrence in medical and health data sets. The analysis of data sets with missing data can lead to loss in statistical power or biased results*”. Eleven works cited problems of missing data with their respective data sets.

The technique most used to overcome this problem was the filling of missing data with the average, used by Sheikhtaheri et al. [22], Sun et al. [23] and Lee et al. [27], followed by the filling with the most frequent data of such attributed, which was used by Sheikhtaheri et al. [22] and Podda et al. [25]. Typically, mean values are used for continuous variables, while most frequent data is more used for categorical values.

Alshwaish et al. [33] and Sun et al. [23] used another technique that fill the missing data with a value not used by that attribute. For example, the weight attribute is filled with the value -1, and the smoking attribute (that accepted 0 for no and 1 for yes) is filled with value 2.

Some other works decided to remove the records that presented this problem (Shukla et al. [10] and Podda et al. [25]), but Mboya et al. [12] removed only the columns that contained a large number of missing data and Cooper et al. [28] removed the records that contained more than 30% missing data. However, both did not report what was done with missing data in records or columns with little missing data.

Valter et al. [29] and Hsu et al. [24] did not describe the strategies used to circumvent the problems with missing data.

It is worth mentioning that the same work can use different techniques to overcome the missing data problem, as was the case of Sheikhtaheri et al. [22], which used the mean for continuous data and more frequent values for boolean or categorical data. Podda et al. [25]) removed all records that contained missing data in the training phase and filled the missing data in the testing phase was filled in with the most frequent values.

#### Attribute selection

Attribute selection is widely applied to reduce the dimensionality of problems and at the same time, according to Remeseiro et al. [38], it can also reduce measurement cost and improve model learning, impacting its performance. Most works (12 out of 18) did not describe the number of attributes of their original data set, but most of them cite how many attributes were selected for training.

Nine works [10, 12, 15, 21, 22, 25, 26, 28, 29] performed some technique for the selection of attributes, while five did not mentioned and four of them did not make clear if they used any attribute selection technique.

The use of an specialist in the area of interest was one of the techniques used by [21, 22, 25]. Commonly, this technique is used to validate the attributes selected by some other computational technique, but it can also be used individually. Using literature as a basis for choosing attributes was also a technique used by [10, 22].

Mboya et al. [12] and Koivu et al. [15] used Random Forest and Logistic Regression algorithms to perform the selection of attributes. This process can be carried out through an univariate evaluation (it uses one attribute at a time to evaluate how significant that variable can be for that problem) or through an additional evaluation (where the most significant variables are grouped until the addition of new variables does not further improve predicted outcomes) [39].

Other correlation methods were also used, such as Pearson’s correlation by Koivu et al. [15], and correlation-based resource subset selection by Sheikhtaheri et al. [22], which are methods that aim to find a correlation between attributes, that is, measure how much one attribute influences another [40]. These methods are normally used for linear problems.

Statistical tests were also used to select the best attributes, such as the Wilcoxon test, the Mann-Whitney nonparametric test and the Chi-square test. These type of technique typically analyze attributes individually and assess their statistical importance [39].

Three works did not specify nor the attributes of the data set, neither the final attributes: [14, 26, 33]. Having these information is crucial for reproductibility of the work, and the lack of them difficult a fair comparison and discussion of their results.

Saravanou et al. [30] performed the biggest reduction of attributes, leaving models with one or two of the 128 attributes from the original data set, a reduction of about 99%; while Batista et al. [31] used all available attributes of the data set (23 attributes).

**Fig. 4:**
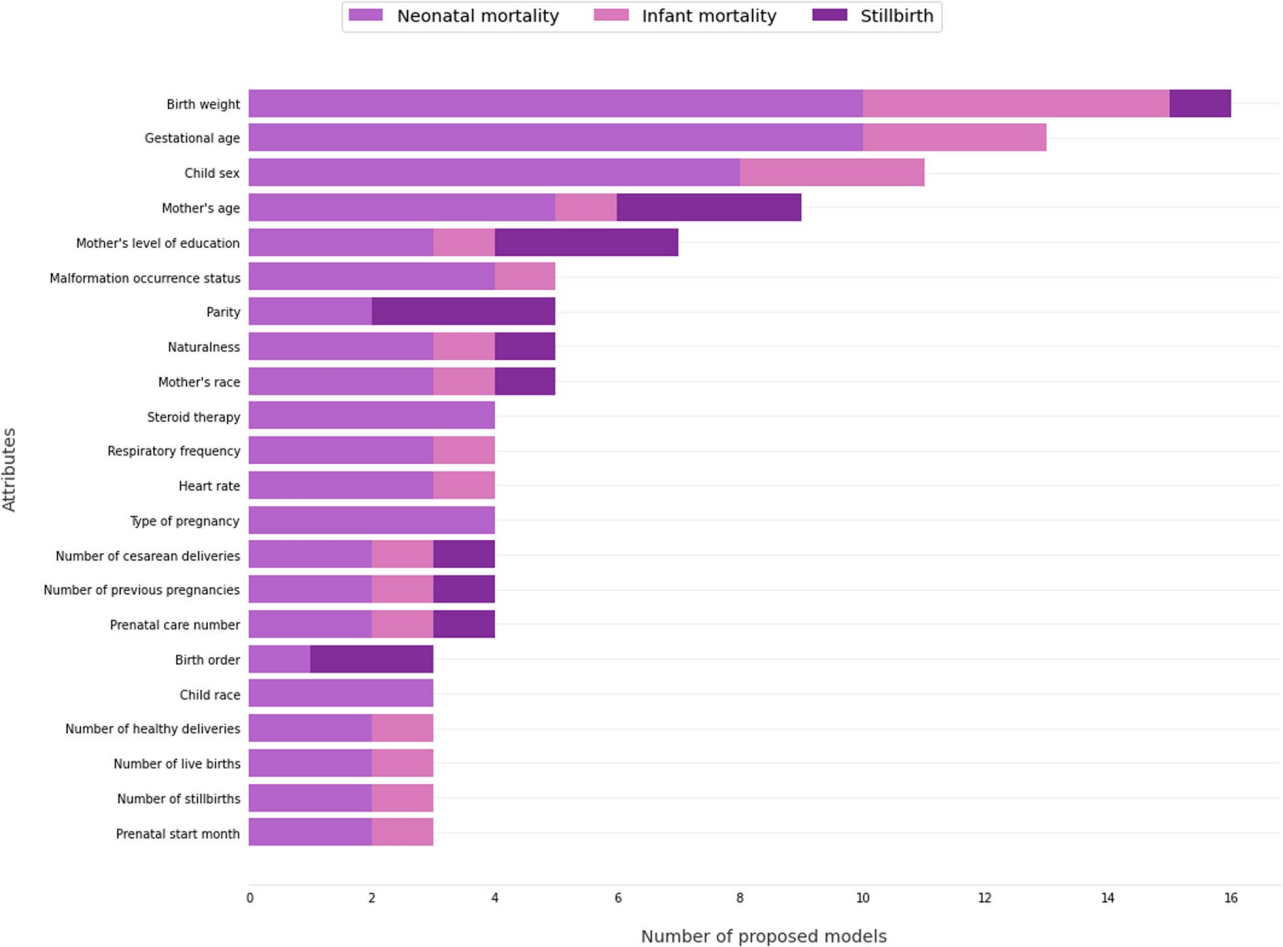
Common features used by primary works, considering fetal, neonatal and infant death

Regarding the attributes used, Fig. 4 presents the most frequent attributes found in the works, separated by the type of mortality.^6^^,^^7^ The attributes birth weight, gestational age and sex of the child were the most frequent with 16, 13 and 11 occurrences in the primary works. Followed by the apgar score, mother’s age, multiple births and mother’s education.

When working with prediction of neonatal mortality, the six most frequent attributes were: birth weight, gestational age, child sex, Apgar score, maternal age and multiple births.

For prediction of infant mortality, the four most frequent attributes are birth weight, gestational age, child sex and Apgar score, followed by multiple deliveries. For the prediction of stillbirths, attributes such as mother’s age, education, and parity are the most frequent, followed by gestational age at enrollment, perinatal mortality cluster,^8^ and the number of prenatal consultations. It is worth mentioning that many attributes regular in neonatal and infant mortality cannot be used when considering stillbirth cases, such as gestational age, since it is information about when the birth occurred. The detection of stillbirths precedes this information.

Some attributes that appeared were related to comorbidities of the mother or child, such as diabetes, sepsis, hypertension and hemorrhage. Other attributes related to sociodemographics, such as mother and child race, mother’s job, mother’s marital status, and smoking. Several attributes are also related to previous pregnancies, such as number of previous pregnancies, number of stillbirths, number of live births, number of cesarean sections; and the current pregnancy, such as prenatal care, type of delivery, height at birth, birth order, birth companion.

### What machine learning and deep learning techniques are being used in researches related to the classification of mortality?

#### Classification problem

Of the 18 works selected in this SLR, 15 of them solved a binary classification problem, one work focused on multiclass classification and two works proposed models for both binary and multiclass classifications.

Of the binary classifications, all works are related to mortality and alive (neonatal mortality and alive, or infant and alive, or stillbirth and alive, or perinatal and alive), while the three multiclass classification works used another perspective. Saravanou et al. [30] considered six different classes: died < 1 h, died between 1 and 23 h, died between 1 and 6 days, died between 7 and 27 days, died between 28 and 365 days and alive. AlShwaish et al. [33] classified risk levels of mortality, considering four classes, from minor to extreme. And Koivu et al. [15] classified into early stillbirth, late stillbirth and non-stillbirth, as shown earlier in Table 1.

#### Modeling techniques

Machine learning was the most common modeling technique found among the primary works of this SLR, being proposed by 16 of the 18 works [10, 12, 14, 15, 21, 22, 24–33]. Deep learning models in turn were proposed by four works [15, 20, 23, 33]. In addition to machine learning and deep learning techniques, 11 works also presented Logistic Regression models [10, 12, 14, 15, 23, 25, 26, 28, 31–33].

**Fig. 5:**
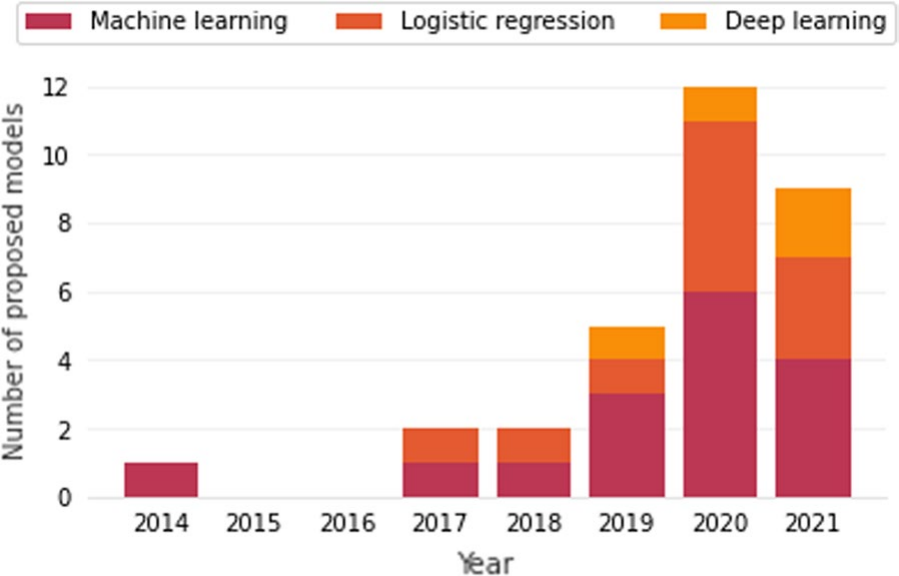
Type of modeling technique by the year of work publication

When analyzing Fig. 5, one can note that deep learning models appeared from 2019 on wards, showing that there may be a large field of search in relation to these models.

**Fig. 6:**
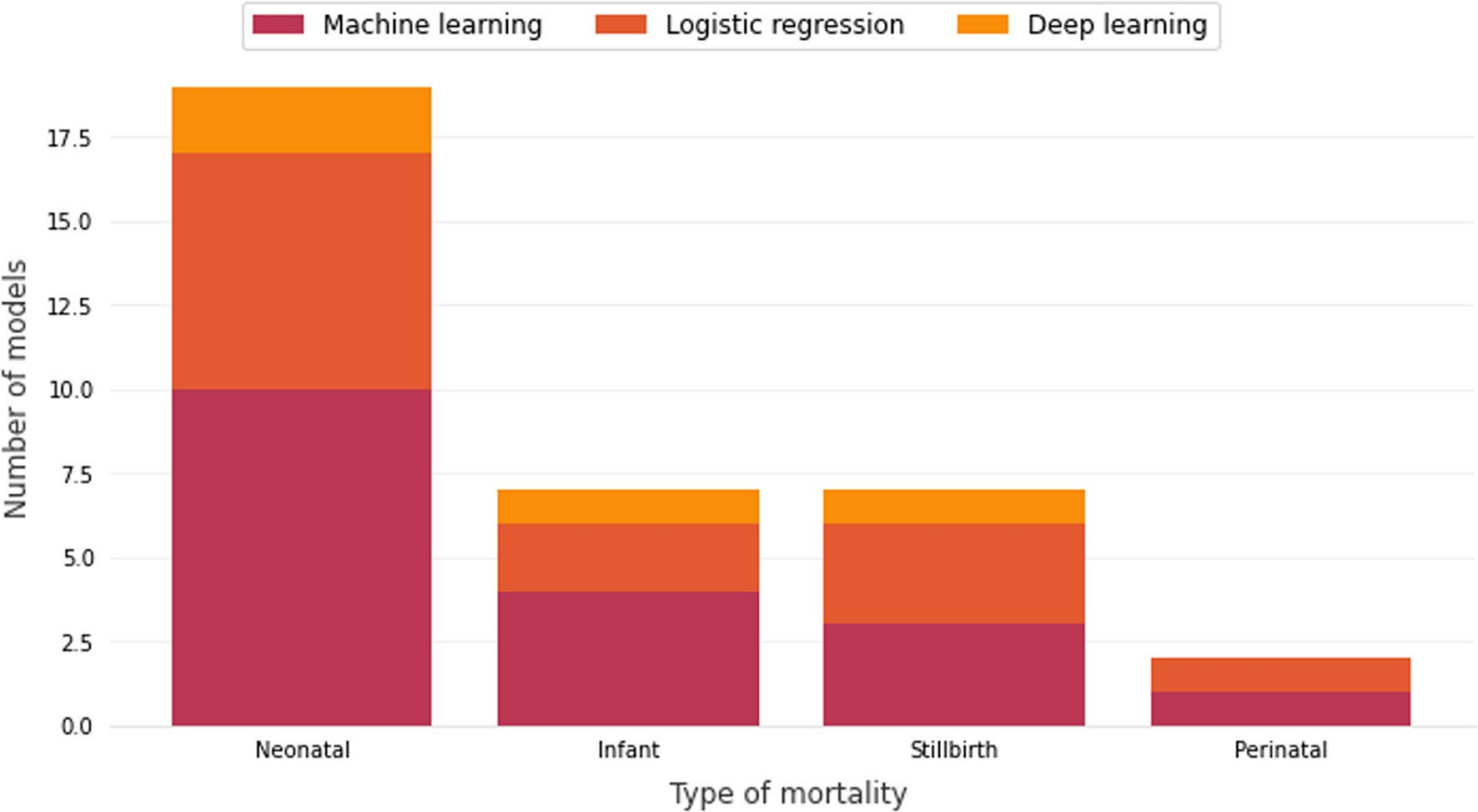
Number of primary works and the modeling technique that was proposed based on the type of mortality classification

Figure 6 shows the number of works and the modeling technique that was proposed based on the type of mortality classification.

Regarding the neonatal mortality works, ten machine learning models were proposed [10, 21, 22, 24–29, 31], two deep learning models were proposed [20, 23], and seven logistic regression models were proposed [10, 23–26, 28, 31]. Even though infant mortality was the focus of more works than stillbirth, the total of proposed models was the same, seven for each.

**Fig. 7:**
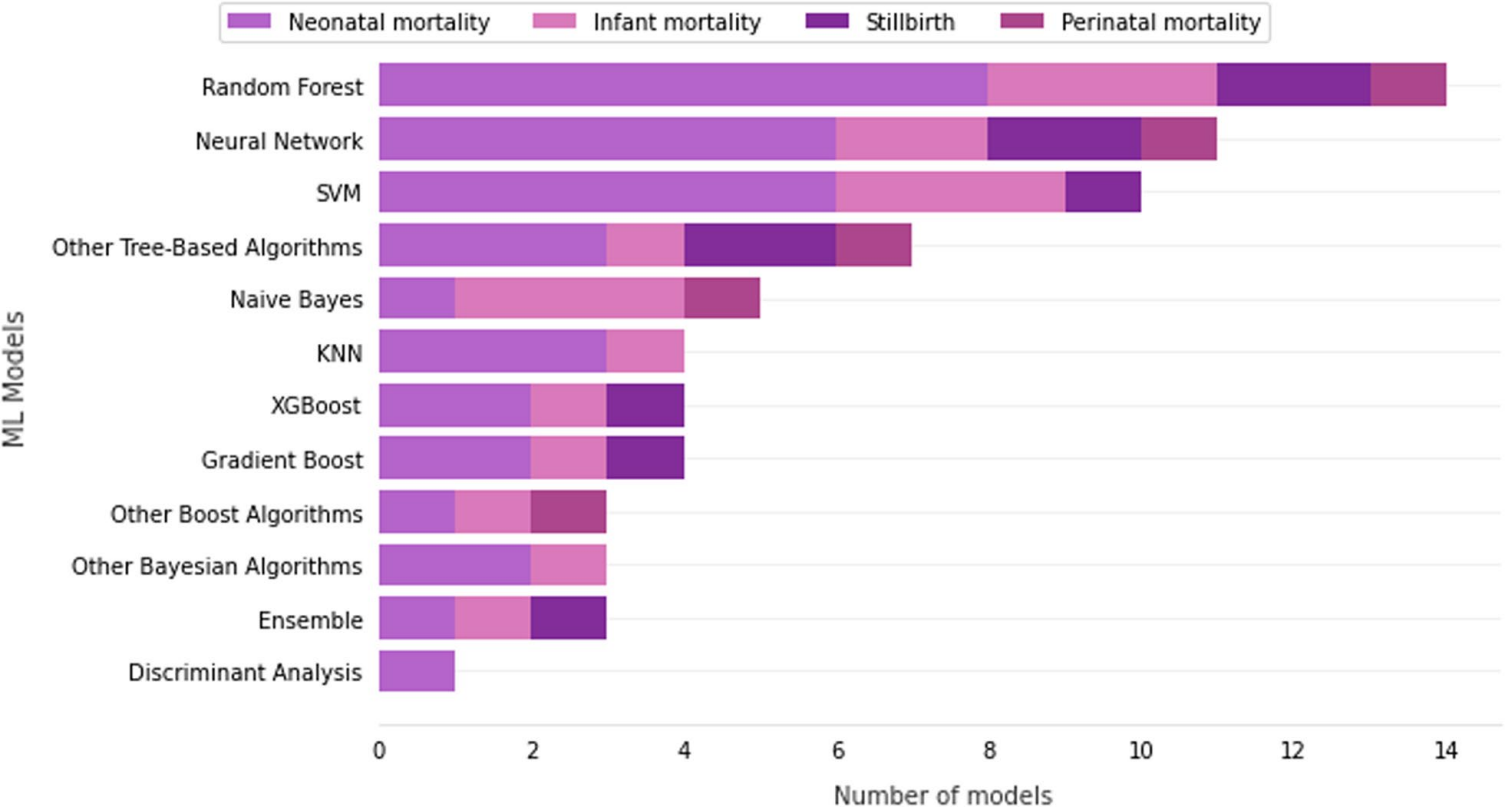
Machine learning techniques and number of proposed models found in primary works

Figure 7 presents the type of machine learning technique and the number of works that proposed them. The most common machine learning model among the primary works was the Random Forest, with 14 proposals, followed by the Neural Network and Support Vector Machines (SVM) with 11 and 10 proposals, respectively. In addition to these models, other common machine learning models are Naive Bayes, K-Nearest Neighbors (KNN), XGBoost, Gradient Boost and ensemble models.

**Fig. 8:**
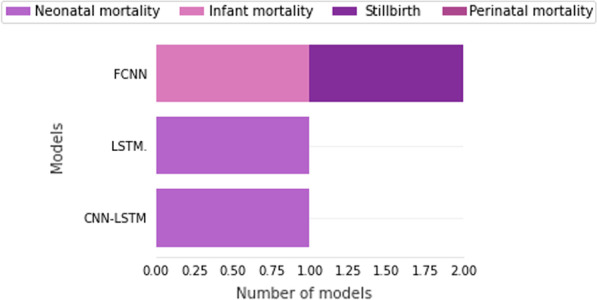
Deep learning techniques and number of proposed models found in primary works

Figure 8 shows the deep learning models proposed by type of mortality. As mentioned before, deep learning models were found only in four works. The Fully Con- nected Neural Network (FCNN) was the most frequent model, proposed by two works, followed by the Long Short-Term Memory (LSTM) model and the joint model called CNN-LSTM. The CNN-LSTM unites two deep learning models (Con- volutional Neural Network (CNN) and LSTM) into a single model.

As observed, Random Forest and FCNN were the most common machine learning and deep learning models proposed to predict mortality, respectively. As primary works are handling with tabular data, the usage of Random Forest is expected since they are Valter et al. [29], Hajipour et al. [32], Shukla et al. [10], Malacova et al. [14], Sheikhtaheri et al. [22], Podda et al. [25], Jaskari et al. [26], Mboya et al. [12], AlShwaish et al. [33], Lee et al. [27], Cooper et al. [28] and Hsu et al. [24]. However, we would like to highlight other tree-based algorithms that have been gained attention in literature, such as XGBoost and Gradient Boost. They are Saravanou et al. [30], Shukla et al. [10], Malacova et al. [14], Podda et al. [25], Batista et al. [31], AlShawaish et al. [33] and Hsu et al. [24].

#### Hyperparameter optimization

According to Yu et al. [41], an expert can provide a consistent set for model initialization parameters (hyperparameter), but in most cases, these parameters may not be optimal. Also, according to Yu et al. [41], performing the adjustments of these hyperparameters is a primordial phase in the entire process of training machine learning and deep learning models.

Of the primary works of this SLR, 10 of them (more than half) did not applied any hyperparameter optimization. Of the 8 works that used it, 6 used a technique called Grid Search [14, 24, 25, 27, 29, 30]. Grid Search is a traditional technique that uses an exhaustive search within a given limited search space [42]. That is, it is necessary to define a range of values for specific hyperparameters, which, in a grid format, is evaluated one by one, in search of the best combination.

Batista et al. [31] used the Bayesian algorithm, that in simple terms, creates an approximate function of the objective function to find the promising regions for the best hyperparameter. With this, its search field is very limited, but faster in the search for parameters [43]. Jascari et al. [26] used nested cross-validation to estimate the generalization performance of selected parameters.

#### Model validation

There are different ways to calculate the classification error of a model and the most popular is the k-fold cross validation. According to Rodriguez et al. [44], in this approach, the data set is divided into *k* folds and the target model is trained using $k-1$ folds. The error value of the training phase is calculated by testing the model with the remaining fold (test fold) and the final error of the model is the average value of the errors calculated in each iteration.

Most of works (14 of 18) applied the k-fold cross-validation approach to validate their models; of these 14 works, nine of them used $k=10$, one used $k=8$ and four used $k=5$. We highlight that, according to Fushiki [45], “*k-fold cross validation has an upward bias, and the bias may not be neglected when k* *is small*” and therefore, it is important to analyze the value of *k* according to the size of the data set available for the study.

### How is the performance of machine learning and deep learning models evaluated in the classification of mortality?

#### Evaluation metrics

Choosing the appropriate way to evaluate the proposed models plays a critical role in the process of obtaining the ideal classifier; that is, the selection of the metrics pertinent to the problem is a key to a better evaluation of the models and to detect the best classifier for the proposed trial [46].

Most evaluation metrics in classification problems are based on the confusion matrix. As shown in Table 3, a confusion matrix is composed of: True Positive (TP), when the positive class is correctly classified; True Negative(TN), when the negative class is correctly classified; False Positive (FP), when a negative class is classified as positive; and the False Negative (FN), when a positive class is classified as negative.

**Table 3:** Generic confusion matrix

Predicted Values	Actual values	
	Positive	Negative
Positive	TP	FP
Negative	FN	TN

Based on TP, TN, FP, and FN, different evaluation metrics can be defined. The most commonly found metric is the accuracy. Accuracy calculates how often the classifier was correct in its classification, according to the Equation 1:

1 $$\begin{aligned} accuracy = \frac{TP + TN}{TP + TN + FP + FN} \end{aligned}$$

Precision is the metric that calculates how many cases were classified as positive that were actually positive, as shown in Equation 2. It is used when the FP are considered more relevant than FN.

2 $$\begin{aligned} precision = \frac{TP}{TP + FP} \end{aligned}$$

Sensitivity, also known as recall, is the metric that calculates the proportion of actual positives that was correctly classified, as presented in Equation 3. It is used when the FN is considered more relevant than FP.

3 $$\begin{aligned} sensitivity = \frac{TP}{TP + FN} \end{aligned}$$

Opposite to sensitivity, the specificity metric is the proportion of negative cases correctly classified and it is calculates according to Equation 4).

4 $$\begin{aligned} specificity = \frac{TN}{TN + FP} \end{aligned}$$

The F1-score metric is the harmonic mean between precision and sensitivity, calculated as shown in Equation 5. This metric gives greater weight to lower numbers, so if one of the two metrics has a low value, the result will be similarly low. This harmonic mean is advantageous when the objective is to seek a balance between these two metrics.

5 $$\begin{aligned} F1{\text {-}}score = 2 \times \frac{precision \times sensitivity}{precision + sensitivity} \end{aligned}$$

These are the most well-known and used metrics based on the confusion matrix. Table 4 presents the metrics used in the primary works. Sensitivity and accuracy appears in 11 works [12, 14, 20–22, 24, 26, 27, 29–33], specificity in nine [12, 14, 20–22, 26, 27, 31, 32], F1-score, and precision in seven [14, 22–24, 26, 30–33].

**Table 4:** Metrics by selected works

Work	Metrics
Valter et al. [29]	Accuracy, AUC ROC
Hajipour et al. [32]	Accuracy, Precision, Specificity, Sensibility, F1-score, AUC ROC
Saravanou et al. [30]	Precision, Sensibility, AUC ROC
Baker et al. [20]	Accuracy, Specificity, Sensibility, AUC ROC, AUPRC
Cerqueira et al. [21]	Accuracy, Specificity, Sensibility, AUC ROC
Shukla et al. [10]	AUC ROC
Malacova et al. [14]	Accuracy, Precision, Specificity, Sensibility, AUC ROC
Sheikhtaheri et al. [22]	Accuracy, Precision, Specificity, Sensibility, F1-score, AUC ROC
Podda et al. [25]	AUC ROC
Batista et al. [31]	Precision, Specificity, Sensibility, F1-score, AUC ROC, AUPRC
Jaskari et al. [26]	Accuracy, Precision, Specificity, Sensibility, F1-score, AUC ROC
Mboya et al. [12]	Accuracy, Specificity,Sensibility, AUC ROC, PPV, NPV, p-value
AlShwaish et al. [33]	Accuracy, Precision, Sensibility, F1-score, AUC ROC
Sun et al. [23]	F1-score, AUC ROC, AUPRC, PPV, NPV
Koivu et al. [15]	AUC ROC, TPR at 10% FPR
Lee et al. [27]	Accuracy, Specificity, Sensibility, AUC ROC
Cooper et al. [28]	AUC ROC, MSE
Hsu et al. [24]	Accuracy, F1-score, AUC ROC

Area under the ROC curve (AUC ROC) was the metric that appeared in all primary works of this SLR, showing its importance in evaluating classifiers models. To understand this metric, let’s first understand the receiver operating characteristic curve (ROC curve). The ROC curve is a two-dimensional graph that balances the benefits, True Positive Rate (TPR) (sensitivity), and the costs, False Positive Rate (FPR), which is calculates as shown in Equation 6:

6 $$\begin{aligned} FPR = 1 - specificity \end{aligned}$$

However, using the ROC curve to compare different classifiers is not easy, so the AUC ROC [47] metric is used. The AUC ROC is the area under the ROC curve, which is bounded between 0 and 1. A model with an AUC ROC close to 1 has a good performance rating, while a model with an AUC ROC close to 0 is rated as poor performance.

Other metrics were also used, however, less frequently, such as the Area under the precision-recall curve (AUPRC). The AUPRC is used in Baker et al. [20], Batista et al. [31], and Sun et al. [23] and it is a variance of the AUC ROC, a more appropriate metric for unbalanced class databases with a problem configured in predicting the positive class since it uses precision-recall curves [48].

Mboya et al. [12] and Sun et al. [23] used Positive Predictive Value (PPV) (precision) and Negative Predictive Value (NPV) metrics. The NPV metric is the inverse of precision, which aims to verify from negative values, which were classified as negative [49], its calculation is defined by Equation 7:

7 $$\begin{aligned} NPV =\frac{TN}{TN+FN} \end{aligned}$$

#### Metrics for imbalanced data sets

When working with imbalanced data sets, we need to use metrics that do not bias the evaluation due this imbalance as they can present overly optimistic results. According to Chicco et al. [50], accuracy and AUC ROC are metrics sensitive to the imbalance of classes, while precision, sensitivity, specificity, and F1-score are metrics that do not analyze all the confusion matrix values, which can lead to unfair observations of the results.

**Fig. 9:**
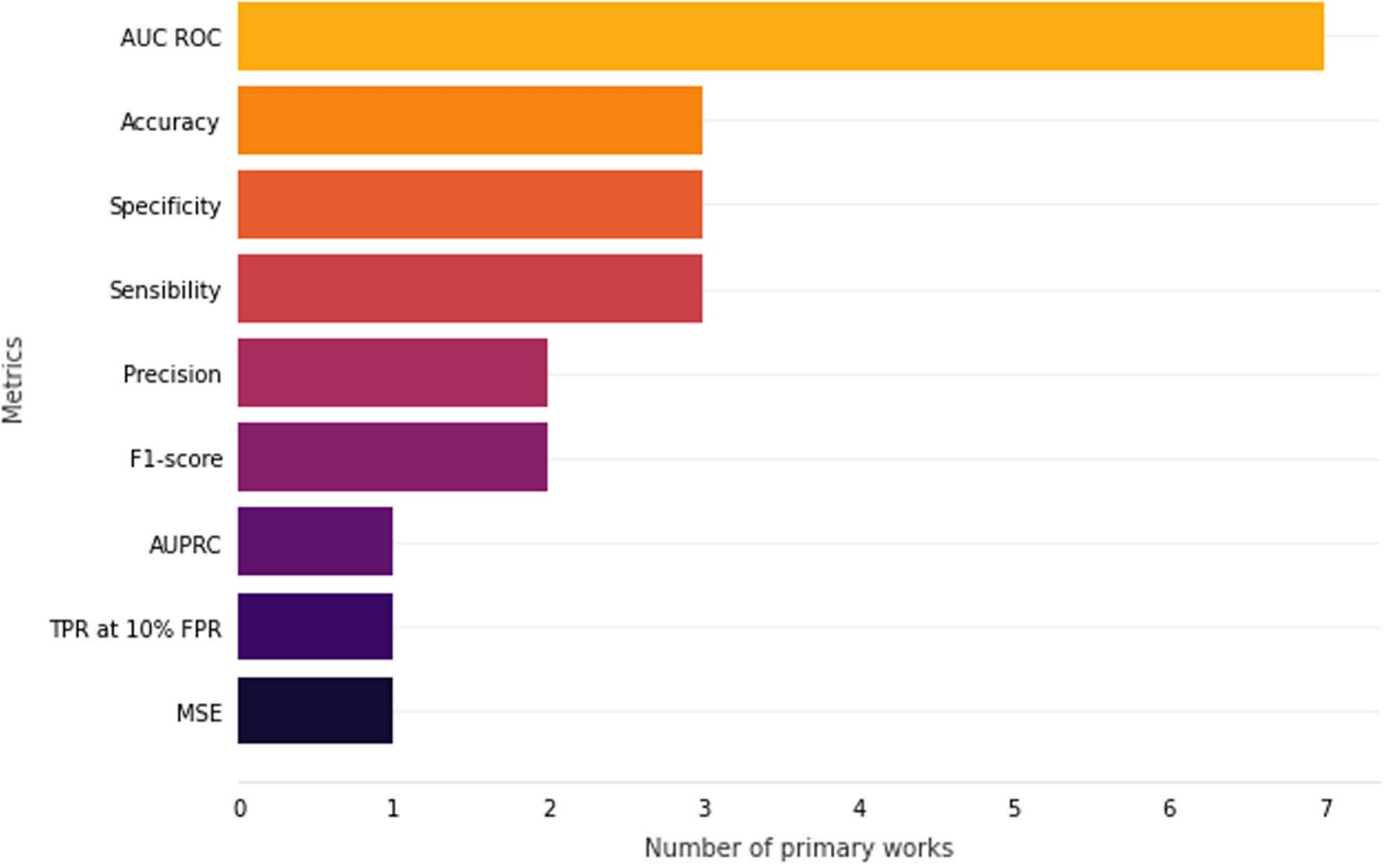
The most common metrics used to evaluate model performance when working with imbalanced data set

Figure 9 shows the metrics used by works that trained their models with imbalanced data sets. All seven works that used imbalanced data set used the AUC ROC metric; three used accuracy [14, 21, 24], which is one of the most sensible metric when working with imbalanced classes. The AUPRC metric, which according to Chicco et al. [50], is the more robust metrics to evaluate a model performance when handling imbalancing, was only used by Batista et al. [31].

#### Statistical tests

The use of statistical tests when developing machine learning models was already mentioned in subsection , when describing techniques to deal with feature selection. Here, the use of statistical tests are focused on the definition of the best model based on the evaluation metrics.

Four works used statistical testing to evaluate and choose their best models. Podda et al. [25], Mboya et al. [12], and Hsu et al. [24] used the DeLong test to evaluate their models. DeLong test verifies if there is a significant difference between the AUC ROC results of the two models [51].

Shukla et al. [10] used the pairwise t-test, that is to compare two population means where there are two samples in which observations from one sample can be paired with observations from the other [52].

## Discussion

The use of predictive models to estimate stillbirth risk may benefit women during prenatal testing. Trudell et al. [53] described the risk of stillbirth starting at 32 weeks of gestational age. It has been seen that non-stress testing done during prenatal care can prevent 6 to 8 stillbirths per 10,000 pregnancies. Thus, showing the importance of predictive models for predicting and avoiding stillbirths during gestation.

Complementary to the prediction, the definition of most relevant predictors is also a relevant contribution. For instance, in Western Ethiopia [54], a study highlighted some predictors of neonatal mortality based on local data. Conditions such as age less than 20 years, primiparous, complications during pregnancy and childbirth, prenatal visits, small size neonates, home birth and gestational age less than 37 weeks are predictors of neonatal mortality. Predictive data was important to knowledge the reasons about the local neonatal mortality rate increased during recent years. Circumstances such as low coverage of health services in the region, low access and use of obstetric services and early pregnancy contribute to increased mortality rates.

The prediction of neonates who are at risk of death can help health professionals to provide early treatment, increasing the chances of survival and minimizing the morbidity rate [22]. Recent studies showed that model predictions based on multiple factors such as gestational and infant are more accurate in estimating than those based only in insulated factor, such as gestational age. Prenatal and postnatal interventions can reduce neonatal mortality and morbidity, and multifactorial based models would optimize such care in practical use [55, 56]. It is essential to have enough data to analyze several factors and then produce more assertive predictive models that can be applied in the health system.

In this SLR, we found data sets with different sizes (varying between 293 to over 31 million records), number of attributes (from 26 to 128) and with missing values and imbalanced classes. According to an UNESCO report [1], “*poor data availability and quality require innovative methodological work to understand the global picture of stillbirths*”. And it is true not only for stillbirths but also for perinatal, neonatal and infant mortality. Some authors have put efforts to minimize the issues related to the quality of their data sets and traditional techniques, such as the average value was used to fill missing data; ROS and SMOTE were used to balancing classes. However, there are many other techniques that can be applied in order to improve the quality of the data before the model training. For instance, for high-dimensional data, one can apply different types of dimensionality reduction in order to reduce redundancy and noise. According to Huang et al. [57], these techniques can also reduce the complexity of learning models and improve their classification performance.

The proposal of deep learning models to classify mortality is still in early stages, having only four works published at the time of writing this systematic review. This is not so surprising, since machine learning models are more efficient to handle tabular data (that is the common data type used for mortality classification), while deep learning are good models to recognize objects in an image based on the spacial relationship of the pixels. Based on this fact, it is possible to improve the performance of deep learning models when using tabular data by transcribing the tabular data into images. Zhu et al. [58] state that the data set features can be arranged into a 2D space, using techniques such as feature similarity and feature distance [58, 59]. With this, deep learning models would learn tabular data using their strengths.

It is important to highlight that health issues found in high income countries (HICs) are very different from those in low and middle incomes countries (LMICs). Computational models are presented as a low-cost (implementation and maintenance) but high accuracy solution, especially for LMICs, since such solutions can be available in an online fashion.

The findings of this SLR are similar to ones found in SLRs about other domains, including the work done by [60], which investigated the use of AI models for clinical diagnosis of arboviral diseases, and [61], which sought models of machine learning in geriatric clinical care for chronic diseases. These conclusions mostly concern the models’ shortcomings and strengths, as well as the pre-processing of the data.

Additionally, maternal mortality is a research area that we would like to highlight for further investigation and as complement of this one. According to Geller et al. [62], maternal mortality “*is used globally to monitor maternal health, the general quality of reproductive health care, and the progress countries have made toward international development goals*”. In a quickly investigation, we found only few recent (and incipient) works that focus on maternal mortality [63, 64], showing that there are many research opportunities to contribute in this area.

This SLR is also essential for the development of new researches. We have analyzed and discussed several aspects of machine and deep learning development, so readers can use this work as a good kick off to choose the best strategies for solving their problems and designing their methodology in a more robust way, facilitating scientific reproducibility.

## Conclusions and next steps

Mortality during pregnancy or during the first few weeks of life may reveal how well pregnant women and their newborns are cared for by health institutions. Due to its feasible operational cost, utilizing technology to assist medical professionals during and after pregnancy has shown to be a powerful ally for enhancing both public health and the quality of prenatal care.

On the other hand, the computational models created based on data from a specific location are particularly generalist only for that region, making them difficult to apply to another location without modifications. In other words, countries with limited resources may struggle with a lack of data or with data of low quality, which has a direct impact on the performance of the computational models.

In this work, we found 18 articles that classified unfavorable pregnancy outcomes-such as stillbirth, perinatal, neonatal, and/or infant mortality-using machine learning and/or deep learning. We discovered that the classification of neonatal death was the most researched, while the parameters birth weight, gestational age, child’s gender, and mother’s age were most frequently employed in studies. The random forest machine learning model was the most commonly suggested model, while the AUC ROC assessment metric was most frequently utilized to rate the models.

With this work, we were able to identify several research gaps and areas for further investigation, such as maternal mortality and morbidities, but more importantly, we offered several potential approaches for individuals wishing to pursue these goals and use these kinds of data.

## Data Availability

The datasets used and/or analysed during the current study available from the corresponding author on reasonable request.

## Abbreviations

AI: Artificial Intelligence
AUC ROC: Area under the ROC curve
AUPRC: Area under the precision-recall curve
CNN: Convolutional Neural Network
FCNN: Fully Connected Neural Network
FN: False Negative
FP: False Positive
FPR: False Positive Rate
HICs: high income countries
ICD-10: International Classification of Diseases
KNN: K-Nearest Neighbors
LMICs: low and middle incomes countries
LSTM: Long Short-Term Memory
MDGs: Millennium Development Goals
NICU: Neonatal Intensive Care Unit
NPV: Negative Predictive Value
PPV: Positive Predictive Value
ROC curve: receiver operating characteristic curve
ROS: random oversampling
RQ: research questions
RUS: random undersampling
SDGs: Sustainable Development Goals
SLR: Systematic literature review
SMOTE: Synthetic Minority Oversampling Technique
SVM: Support Vector Machines
TN: True Negative
TP: True Positive
TPR: True Positive Rate
UN: United Nations
USA: United States of America
WHO: World Health Organization

## Footnotes

1 https://IEEExplore.ieee.org/Xplore/home.jsp.

2 https://pubmed.ncbi.nlm.nih.gov.

3 https://dl.acm.org.

4 https://link.springer.com.

5 https://www.scopus.com.

6 The attributes that appear only once were removed from the figure.

7 Perinatal mortality is not is in the Figure because the work that addresses this mortality has not described its attributes.

8 This attribute is the mortality rate in previous years of that location to influence the forecast of new cases.
